# Cross-sectional imaging and cytologic investigations in the preoperative diagnosis of parotid gland tumors – An updated literature review

**DOI:** 10.17305/bjbms.2020.5028

**Published:** 2021-02

**Authors:** Sebastian Stoia, Grigore Băciuț, Manuela Lenghel, Radu Badea, Csaba Csutak, Georgeta Mihaela Rusu, Mihaela Băciuț, Tiberiu Tamaş, Emil Boțan, Gabriel Armencea, Simion Bran, Cristian Dinu

**Affiliations:** 1 Department of Maxillofacial Surgery and Implantology, Faculty of Dentistry, “Iuliu Hațieganu” University of Medicine and Pharmacy, Cluj-Napoca, Romania; 2 Department of Radiology, Faculty of Medicine, “Iuliu Hațieganu” University of Medicine and Pharmacy, Cluj-Napoca, Romania; 3 Department of Medical Imaging, Faculty of Medicine, “Iuliu Hațieganu” University of Medicine and Pharmacy, Department of Medical Imaging, „Prof. Dr. Octavian Fodor” Regional Institute of Gastroenterology, Cluj-Napoca, Romania; 4 Department of Pathology, Emergency County Hospital, Cluj-Napoca, Romania

**Keywords:** Imaging, fine needle aspiration biopsy, core needle biopsy, diagnosis, parotid gland tumors

## Abstract

An accurate preoperative diagnosis of parotid tumors is essential for the selection and planning of surgical treatment. Various modern cross-sectional imaging and cytologic investigations can support the differential diagnosis of parotid tumors. The aim of this study was to achieve a comprehensive and updated review of modern imaging and cytologic investigations used in parotid tumor diagnosis, based on the latest literature data. This literature review could serve as a guide for clinicians in selecting different types of investigations for the preoperative differential diagnosis of parotid tumors. Magnetic resonance imaging (MRI) with its dynamic and advanced sequences is the first-line imaging investigation used in differentiating parotid tumors. Computed tomography (CT) and positron emission tomography (PET)-CT provide limited indications in differentiating parotid tumors. Fine needle aspiration biopsy and core needle biopsy can contribute with satisfactory results to the cytological diagnosis of parotid tumors. Dynamic MRI with its dynamic contrast-enhanced and diffusion-weighted sequences provides the best accuracy for the preoperative differential diagnosis of parotid tumors. CT allows the best evaluation of bone invasion, being useful when MRI cannot be performed, and PET-CT has value in the follow-up of cancer patients. The dual cytological and imaging approach is the safest method for an accurate differential diagnosis of parotid tumors.

## INTRODUCTION

Salivary gland tumors account between 2% and 6.5% of all head and neck tumors being most commonly located in the parotid glands, having a wide histological variety. The latest histological classification of salivary gland tumors published in 2017 by the World Health Organization describes 20 malignant and 11 benign histological entities [1,2].

Regarding the frequency and location of parotid tumors, many authors accept the rule of “80”: 80% of salivary gland tumors are located in the parotid gland, 80% are benign tumors, 80% are located in the superficial lobe, and 80% are pleomorphic adenomas (PMA). Warthin tumor (WT) is the second most common parotid tumor (15–30%). PMA is found mainly in middle-aged women, while WT is found often in older men. Malignant parotid tumors are encountered in 20–30% of cases, mucoepidermoid carcinoma being the most common histological type (30%) [1-7].

Benign parotid tumors present as painless, well-defined, mobile lesions, of firm-elastic consistency, with slow growth, without alterations of the overlying skin, remaining asymptomatic for a long period of time. Dysphagia may be present in deep lobe parotid tumors or in cases of parotid tumors with extension in the parapharyngeal space. A significant proportion of malignant tumors exhibit a clinical picture difficult to differentiate from benign tumors, especially in the early stages of evolution. Alarm signs and symptoms of malignant parotid tumors are pain, rapid growth with surrounding tissue invasion, ulcerations of the overlying skin, pathological cervical adenopathy, facial nerve palsy, otalgia, weight loss, and reduced appetite [5,6,8].

Imaging and cytologic investigations are used for the preoperative differential diagnosis of parotid tumors providing information about the tumor cytology, the specific location of the tumor in the gland, and about the relationships with surrounding anatomical structures. Furthermore, these investigations should allow salivary gland cancer staging.

Surgical treatment is the first therapeutic option for parotid gland tumors. Currently, several surgical techniques are accepted, such as partial, total parotidectomy, or extracapsular dissection. Recently, there has been a tendency to limit the extent of surgery in cases of benign parotid tumors. Nowadays, surgery of benign parotid tumors has developed into a conservative operation with low morbidity [2].

Adjuvant radiotherapy is indicated in selected cases of parotid gland malignancies with high-risk features, while chemotherapy may be indicated in certain situations in combination with adjuvant radiotherapy or as a single treatment, for palliative purposes in patients with advanced local disease or metastatic parotid tumors [2,9].

The surgical treatment planning varies according to the histological type of parotid tumor. Surgery for malignant tumors must follow the oncological principles, while benign lesions require less invasive surgical treatment [5,10]. For this reason, preoperative diagnostic classification of parotid lesions into benign or malignant type is of paramount importance. Since biopsy is banned for the parotid territory, because facial nerve injury and permanent salivary fistulae pose a severe risk to the procedure, the preoperative imaging diagnosis supported with cytologic investigation becomes the key element of optimum management.

At present, various modern imaging and cytologic methods are able to differentiate the diagnosis of parotid tumors. Numerous authors have published studies with promising results in this field. To the best of our knowledge, a comprehensive and updated paper, reviewing all the variety of the latest cross-sectional imaging and cytologic investigations used in the parotid gland tumor diagnosis, was not found. This paper aims to achieve an objective and comprehensive review of the modern cross-sectional imaging and cytologic techniques used in parotid tumor diagnosis. The uniqueness of this literature review is represented by its design meant to serve as a guide for clinicians in the selection of different cross-sectional imaging and cytologic investigations, in order to achieve an accurate preoperative differential diagnosis of parotid tumors.

## CROSS-SECTIONAL IMAGING INVESTIGATIONS OF PAROTID GLAND TUMORS

Magnetic resonance imaging (MRI) is a complex, non-invasive investigation, with clinical applications in all medical fields, nowadays undergoing extensive and continuous scientific and technological progress with promising achievements for the future.

MRI has been considered the gold standard imaging technique for parotid gland tumors for decades [11]. The method provides an excellent contrast, spatial resolution and soft tissue characterization with a great variety of signal differences. It can identify and differentiate deep lobe parotid tumors from parapharyngeal space tumors, allowing also the detection of perineural and perivascular spread. The invasion of the skull base, parapharyngeal space, and masticatory muscles can be assessed with MRI. It also detects the extracapsular spread in regional lymph nodes. The intraparotid course of the facial nerve and its relationships with the tumor can be determined with the advanced MRI techniques. The main disadvantages of the method include prolonged time for image acquisition, high cost, susceptibility to motion artifacts, and lower resolution for bone assessment compared to computed tomography (CT). MRI examination cannot be performed in case of claustrophobic patients or those with different medical devices implanted (some models of pacemakers, cochlear implants, or other metallic devices); impaired renal function is a relative contraindication for contrast-enhanced MRI investigation [4,7,12-14]. MRI is mandatory in case of parotid tumors suspected of malignancy, lesions larger than 3 cm or located in the deep parotid lobe or parapharyngeal space [12].

Conventionally, MRI allows the identification and characterization of parotid tumors, having been considered the investigation of choice for a long time. Functional or dynamic MRI, intensely studied today in the differential diagnosis of parotid tumors, includes the following sequences: diffusion-weighted MRI (DWI-MRI) and dynamic contrast-enhanced MRI (DCE-MRI).

The advanced MRI comprises many sequences as follows: diffusion tensor imaging (DTI), dynamic susceptibility contrast (DSC) perfusion-weighted, pseudo/pulsed continuous arterial spin labeling (pCASL), intravoxel incoherent motion (IVIM), track-weighted imaging (TWI), diffusion kurtosis imaging (DKI), and proton MRI spectroscopy (H-MRS). Combining advanced or functional MRI with routine MRI offers promising results in differentiating parotid tumors. With advanced MRI sequences, a series of additional data related to the parotid tumors can be obtained, such as microstructure, vascularity, cellularity, and a detailed assessment of nervous structures [1,15-17].

Espinoza and Halimi, in their technical note article, showed that for the differential diagnosis of parotid tumors MRI examination must include a minimum of sequences, as follows: T1-weighted image (WI) sequence for detecting spontaneous high signal intensity (SI) lesion, T2-WI sequence without fat-saturation for comparing signals between tumors and surrounding parotid tissue, DWI sequence for the evaluation of diffusion restriction (combined with the apparent diffusion coefficient [ADC] map), perfusion-weighted sequence – DCE-MRI, and gadolinium-enhanced fat-saturation T1-WI sequences [18].

An important aspect of the clinical practice is that intra-tumor hemorrhage occurring following the fine needle aspiration biopsy (FNAB) of parotid tumors may interfere with the spontaneous tumor signal in T1 and T2 sequences and may also distort the ADC. Therefore, FNAB should always be performed after MRI investigation [18].

**FIGURE 1:**
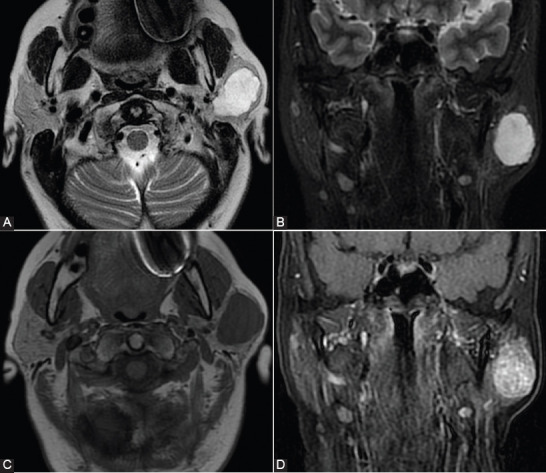
Conventional magnetic resonance imaging appearance in a 60-year-old patient with a left parotid gland pleomorphic adenoma. (A and B) T2-weighted image (WI) sequence and short tau inversion recover (STIR)-WI sequence revealing hyperintense signal. (C and D) T1-WI sequence without contrast and T1 contrast-enhanced sequences showing hypointense signal and homogeneous enhancement.

**FIGURE 2:**
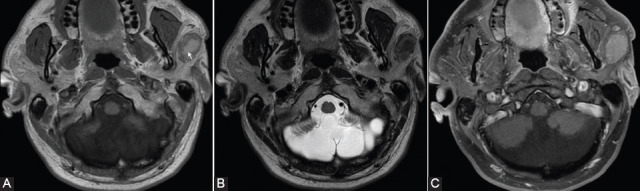
Conventional magnetic resonance imaging appearance in a 50-year-old patient with a Warthin tumor of the left parotid gland. (A) Non-enhanced T1-weighted image (WI) sequence, with inhomogeneous aspect, with central hyperintense area with hemorrhagic transformation (arrow). (B) T2-WI sequence showing a hypointense central area. (C) T1 contrast-enhanced sequences showing enhancement, slightly inhomogeneous aspect.

Conventional MRI investigation allows visualization of parotid glands masses, ensuring proper identification of the tumor margins and extension with the T1-WI sequence. Contrast-enhanced T1-WI is frequently used to evaluate the depth of tumor invasion, but more importantly, it allows the evaluation of tumor enhancement and offers information regarding tumor vascularity [12]. Benign parotid tumors are characterized on conventional MRI by well-defined margins, high SI on T2-WI, and superficial localization. In contrast, malignant parotid tumors demonstrate ill-defined, infiltrative margins, surrounding tissues invasion (sensitivity 68% and specificity 100%), inhomogeneous SI, and T1 hypo-intensity; perineural, perivascular, metastatic lymph nodes. Invasion of deep muscles, bones, or parapharyngeal space are also signs of malignancy [1-3,7,14,15]. Margin assessment showed a sensitivity (Se) of 59–88%, a specificity (Sp) of 79–100%, and an accuracy (Acc) of 73–95%, low SI on T2-WI showed a Se between 18.7 and 73%, a Sp of 93–100%, and an accuracy of 70–78.3% [5,12,19,20]. Perineural invasion showed a Se of 19%, a Sp of 93%, and an accuracy of 74% [19], and surrounding tissue infiltration had a Se of 68% and a Sp of 100% [5] in differentiating benign versus malign parotid tumors. The specific MRI signs for PMA are increased T2-WI SI, marked enhancement and lobulated contours, while the absence of enhancement on post-contrast T1-WI (Figure 1), central hyperintensity on T2-WI, and cystic appearance are suggestive signs for WT (Figure 2) [7,21].

**FIGURE 3:**
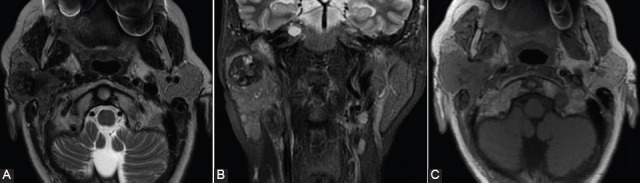
Conventional magnetic resonance imaging appearance in a 70-year-old patient with a malignant tumor of the right parotid gland (carcinoma ex pleomorphic adenoma). (A) T2-weighted image (WI) sequence, (B) short tau inversion recover [STIR], and (C) non-enhanced T1-WI sequences showing an inhomogeneous lesion in the right parotid gland, with infiltrative contour.

Regarding the use of conventional MRI in differentiating benign and malignant parotid tumors, Se, Sp, and accuracy data range between 56.8–87%, 73–100%, and 93–96% (Figure 3) [15,19,22,23].

However, conventional MRI in the differential diagnosis of parotid tumors can sometimes lead to inconclusive results due to the overlaps of the imaging characteristics of benign and malignant tumors. The use of diffusion and perfusion sequences supports and improves the conventional MRI investigation [1,15,23].

## FUNCTIONAL MRI INVESTIGATIONS

DWI-MRI allows the characterization of tissue cellularity, microstructure, and intracellular pathophysiological processes. DWI qualitatively determines the Brownian motion of water molecules between intra and extra cellular compartments of biological tissues. The diffusion of water may undergo modifications in the presence of pathological processes. The inter-cellular conditions, the different tissue type microstructures, and blood microcirculation in the capillary network may influence qualitatively the movement of water molecules. The quantitative evaluation of the water diffusion is possible with the help of the ADC maps generated by the axial DWI images [15,16,24-28]. In order to obtain a numeric ADC value from a region of interest (ROI), the difference of the DWI aspect must be evaluated according to a mono-exponential model, taking into account a minimum of two optimal b-values, which vary on the investigated tissue [26]. Thus, the diffusion of water molecules in a particular tissue type is numerically characterized by the ADC value obtained from a particular ROI [26]. Normal tissue characteristics (extracellular space size, cellular and fiber density, and viscosity) and also tumor tissue features (cell differentiation, density, presence of necrotic or cystic areas, and nucleus/cytoplasm ratio) can be assessed with ADC [25].

Increased cellularity causes a restriction of water molecule diffusion, which leads to lower ADC values (malignant tumors). The low density of cellular components and increased interstitial space will generate high diffusion with high ADC values (benign lesions) [25-27]. Parotid glands tumors can present different ADC values, which can help in establishing a preoperative imaging differential diagnosis. ADC value is one of the most used parameters in differentiating parotid tumors [26].

A large number of studies have been able to differentiate between benign and malignant parotid tumors using DWI-MRI [1,10,15,26,27,29-33], but other studies failed to demonstrate an accurate diagnosis [20,22].

DWI-MRI investigation can distinguish between benign and malignant subtypes of parotid tumors. PMA contains an abundant amount of myxomatous tissue with fluid content within the epithelial glandular regions that determines a reduced restriction of water diffusion. WT contains lymphoid tissue and a large amount of protein-rich fluid, which causes an important restriction of water diffusion and explains the lower ADC values, close to those of malignant tumors. Malignant tumors register lower ADC values due to the high cellularity that causes restriction of water diffusion [1,17]. Therefore, PMA presents the highest ADC value of all parotid tumors, while WT has frequently low ADC values that can overlap with malignant tumors. In most studies, differentiation of PMA from WT, as well as PMA from malignant tumors was successful based on ADC values [15,17,20,25,26,32,34]. Other authors did not obtain significant results regarding the differentiation of WTs from malignant tumors due to the overlap of their ADCs values [25,29,30,32,34]. However, differentiation of WTs from malignant ones, based on ADC maps, was possible in some studies [32,35-37].

Based on the reviewed studies, the ranges of mean ADC values for the most common parotid tumors are presented in Table 1 [15,17,20,22,25,29-32,38].

**TABLE 1:**
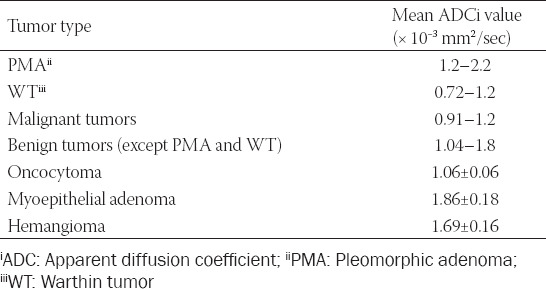
Mean ADC values for parotid tumors

**FIGURE 4:**
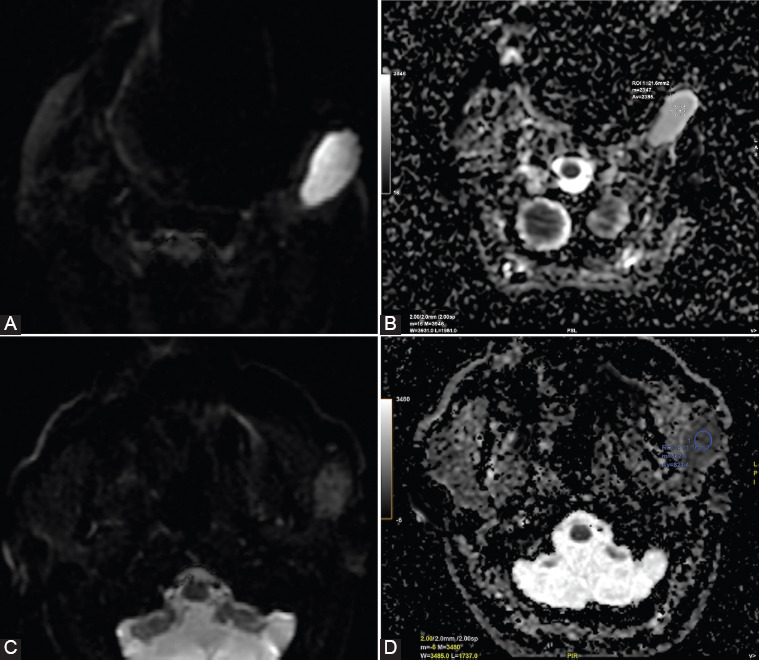
Diffusion-weighted imaging sequences with apparent diffusion coefficient (ADC) map in: (A and B) pleomorphic adenoma with T2-shine through effect and high value of ADC (2,385 × 10^-3^mm^2^/s) and (C and D) Warthin tumor with diffusion restriction and low ADC value (0.821 × 10^-3^mm^2^/s).

The differential diagnosis between PMA and WT on DWI-MRI showed a Se between 84% and 100%, a Sp between 90% and 100%, and an accuracy of 96.2% for the cutoff values between 0.99 and 1.6 (Figure 4).

**FIGURE 5:**
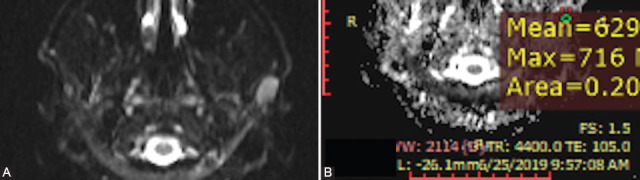
Diffusion-weighted imaging sequences with apparent diffusion coefficient (ADC) map in a 28-year-old patient with primary lymphoma of the left parotid gland, showing diffusion restriction (A) and very low ADC value (B) (× 10^-3^mm^2^/s).

Differentiation of PMA from malignant tumors showed a Se between 82% and 100%, a Sp between 81.3% and 100%, and an accuracy of 97.1% for ADC cutoff values between 1.26 and 1.8. The differential diagnosis of malignant versus benign tumors for the cutoff values between 1.07 and 1.37 recorded a Se between 63.3% and 100%, a Sp between 30% and 71.9%, and an accuracy of approximately 77%. A Se between 91.7 and 92.3%, a Sp between 75% and 100%, and an accuracy between 84.6% and 94.3% were reported for ADC cutoff values between 0.78 and 1.01, for the differentiation of WT from malignant tumors (Figure 5) [15,20,22,25,26,31,32,39].

In most studies, the mean ADC values are calculated from one or two ROIs, located in the most representative tumor area. An image histogram represents a map of pixel intensity values, which shows the number of pixels with the same intensity from the entire image. The ADC parameters used in the histogram analysis are mean value, standard deviation, maximum, minimum, kurtosis, smoothness, and ADC percentiles (10, 50, 90, 99). The use of histogram analysis and ADC measurement of ROIs distributed across all DWI sections to cover the entire tumor may provide additional information and can increase DWI accuracy in parotid tumors diagnosis [27,40].

Razek et al. demonstrated a correlation between ADC values and histopathological features of salivary gland malignancies. Thus, high-grade, higher T stage, and higher N stage tumors showed lower ADC values compared to low-intermediate grade, lower T stage, and lower N stage tumors. Lower ADC values were recorded in the case of malignant tumors with perineural invasion [41].

DWI-MRI sequence can be used in other clinical applications such as differentiating post-treatment changes from tumor recurrences that present lower ADC values, staging and grading parotid gland cancers, the diagnosis of radiotherapy-induced sialadenitis, and not lastly, the diagnosis of lymphomas with parotid localization (lower mean ADC values – 0.55 ± 0.1 × 10^-3^ mm^2^/seconds) [1,17,20,42]. DWI-MRI is indicated for monitoring patients with Sjögren syndrome, as well as for their surveillance for lymphomas with parotid localization, considering the 44 times higher risk of these patients in developing a lymphoma [43].

DCE-MRI contributes to the differential diagnosis of parotid tumors, relying on the identification of different vascular permeability and blood flow between the tumor and the normal parotid tissue. Multiple T1-WI sequences are obtained, for several minutes, from a specific ROI before, during, and after administration of the contrast agent, in order to register the wash-in and the wash-out ratio. The total acquisition time of DCE-MRI sequences, according to most studies, ranges between 105 and 615 seconds [1,15].

The semi-quantitative analysis of DCI-MRI is performed based on the time-intensity curve (TIC), which allows the calculation of SI peak (SI_peak_), time of peak (T_peak_), wash-in, and wash-out ratio (WR). T_peak_ and WR are the most used parameters. T_peak_ represents the time required for the tumor to achieve the enhancement of 90–100% of the contrast agent, from the moment of its administration. T_peak_ correlates with microvascularization, being reduced when microvascularization is increased. The WR is defined as the degree of SI decrease at a given moment after contrast agent administration. Some studies define WR at 3–4 minutes, and others at 5 minutes. The WR is associated with cellular-stromal ratio, increased cellularity leading to a rapid washout [1,15,30,36,44].

Yabuuchi et al. published the first study describing the four types of TICs, based on the WR (30%) and T_peak_ (120 seconds), demonstrating their correlation with histopathological findings and their usefulness in the differential diagnosis between malign and benign salivary gland tumors. The four types of TICs described are type A curve – T_peak_ >120 seconds, type B curve – T_peak_ ≤120 seconds, WR ≥30%, type C curve – T_peak_ ≤120 seconds, WR <30%, and type D curve – flat [37,44]. In their study, the authors observed that PMA had a long T_peak_, WT had a short T_peak_ and high WR, and malignant tumors tended to register a short T_peak_ and low WR [44].

Following the study of Yabuuchi et al. [44], more recent studies on the utility of DCE-MRI in the differential diagnosis of parotid tumors have been published. Their authors used various threshold values for T_peak_ (60–210 seconds) and WRs (10–40%), most of them reporting results that demonstrated the success of investigation in parotid tumors differentiation [15,20,29-31,36,37,45-51].

**FIGURE 6:**
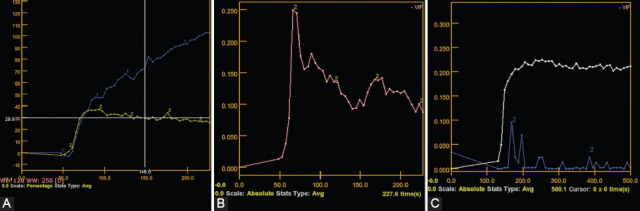
Dynamic contrast-enhanced magnetic resonance imaging. (A) Pleomorphic adenoma (curve type A – curve number 1 on the presented image); (B) Warthin tumor (curve type B); and (C) malignant tumor (curve type C).

After analyzing the studies included in the current review, PMA had a type A TIC curve, WT presented a type B TIC (C or D [30,46]), and malignant tumors registered type C TIC curves (Figure 6) [29,36,37,49,50].

Regarding T_peak_ and WR values, according to each tumor type, the ranges of values recorded in the reviewed studies are presented in Table 2 [15,20,48].

**TABLE 2:**
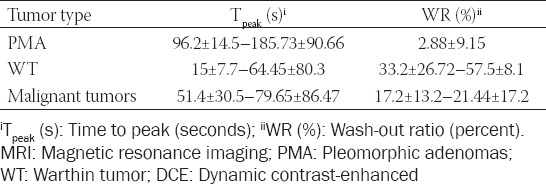
DCE-MRI parameters (T_peak_ and WR) in differentiating parotid tumors

With reference to the DCE-MRI value in the differentiation of the benign and malignant tumors, the following value ranges are presented in the literature: Se of 71–95%, Sp of 76–97.4%, positive predictive value (PPV) of 38–97.4%, negative predictive value (NPV) of 89–99%, and overall accuracy of 79–96.5% [20,29,31,36,37,46]. According to Razek et al., using DCE-MRI, an 84.6% accuracy in the differentiation of PMA from WT was demonstrated and an accuracy of 98% for the differentiation between WT and malignant tumors [31].

Zhu et al. demonstrated that the differential diagnosis between benign lymphoepithelial lesions and mucosa-associated lymphoid tissue lymphoma of the parotid glands can be done with the help of DCE-MRI parameters and TIC curves [47].

Quantitative analysis of DCE-MRI can be performed by following the contrast agent motion between tissue compartments based on perfusion parameters such as K_trans_ (volume constant of transfer from blood plasma and the extracellular-extravascular space [EES]), K_ep_ (diffusion rate constant from the EES to plasma), and V_e_ (EES fractional volume). K_trans_ is in relation to the vascular permeability and K_ep_ is associated with the wash-out phase [24]. Quantitative analysis detects tumor angiogenesis [15,50].

**FIGURE 7:**
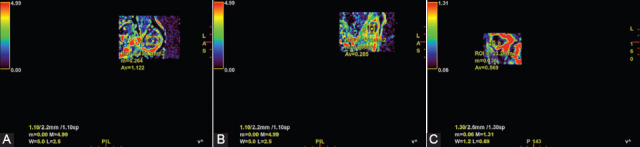
K-trans values in (A) pleomorphic adenoma (high value – 1.122 min^-1^), (B) Warthin tumor (very low value – 0.285 min^-1^), and (C) malignant tumor (intermediate value – 0.569 min^-1^).

According to Zheng et al. and Xu et al., after parotid gland tumors evaluation using the quantitative analysis of DCE-MRI, the following values were obtained for: malignant tumors – K_trans_ 0.327 ± 0.030, K_ep_ 0.784 ± 0.064, V_e_ 0.445 ± 0.025, PMA – K_trans_ 0.217 ± 0.036, K_ep_ 0.567 ± 0.048, V_e_ 0.549 ± 0.278, and WT – K_trans_ 0.464 ± 0.036, K_ep_ 1.806 ± 0.111, V_e_ 0.272 ± 0.013. The same authors reported that for the cutoff values of K_trans_ < 0.435, K_ep_ < 1.118, and V_e_ > 0.315, used for differentiating malignant tumors from benign ones, the Se, Sp, PPV, NPV, and accuracy were 70%, 96%, 82%, 93%, and 92%, respectively. TIC pattern combined with K_ep_ and V_e_ parameters successfully differentiate malignant tumors from PMA, with the cutoff values of K_ep_ > 0.555 and V_e_ <0.605. The values of 90%, 74%, 69%, 92%, and 80%, respectively, were obtained in terms of Se, Sp, PPV, NPV, and accuracy [36,50] (Figure 7).

Combining conventional MRI with DWI and DCE-MRI for the differential diagnosis of parotid tumors the diagnostic value of the method may increase with an overall accuracy of up to 96% [15,29,35,37,45,50].

## ADVANCED MRI

DTI allows the quantitative evaluation of the magnitude and directionality of the micro-movement of water molecules, being able to distinguish between different cellular compartments. Fractional anisotropy (FA) and mean diffusivity (MD) are the most common parameters of DTI. MD represents the mean tensor diffusivity in the three-dimensional space, being equivalent to the ADC parameter. FA is associated with the structural orientation of tissues, reflecting the directionality of the diffusion of water molecules. MD values correlate inversely with tumor cellularity and FA values are directly correlated with tumor cell number and histological grade [15,17,34,52,53]. Takumi et al., Razek et al., and Yu et al. demonstrated the utility of DTI in the differentiation of malignant and benign parotid tumors [34,52,53]. Razek et al. showed in his study that FA and MD values differ significantly between parotid gland tumors [52].

DSC perfusion-weighted allows hemodynamic characterization of salivary gland tumors by following the first passage of the contrast agent through the tissues, with the help of echo-planar imaging; the data acquisition can be performed before the arrival of the bolus. Razek et al. recorded statistically significant differences between parotid tumors using DSC-MRI. They presented an accuracy of 91.7% in differentiating malignant from benign tumors, a 98% accuracy in differentiating WTs from malignant lesions, and an 84.6% accuracy in the differential diagnosis of PMAs from WTs [31].

pCASL is a non-invasive perfusion method that relies on measuring proton magnetization in arterial blood to assess tumor blood flow (TBF). The intrinsic state of arterial blood water is used as a tracer. The technique provides quantitative information about the tumor vascularization based on TBF, without using a contrast agent [10,37]. Razek et al. presented significant differences between mean TBF values for malignant tumors (0.66 ± 0.1 × 10^-3^ mm^2^/seconds) compared to benign lesions (1.28 ± 0.4 × 10^-3^ mm^2^/seconds) [54]. Yamamoto et al. were able to differentiate WTs from PMAs using TBF values of pCASL-MRI: 0.85 × 10^-3^ mm^2^/seconds for WT and 1.57 × 10^-3^ mm^2^/seconds for PMA [49].

IVIM, with the help of a single DWI acquisition, allows precise estimation of water diffusion at the level of the capillary network. Using multiple b-values, IVIM allows the generation of a bi-exponential model as well as the diffusion and perfusion maps, without using the contrast agent. The combined use of DCE-MRI and IVIM can provide new features potentially useful in the differential diagnosis of parotid tumors [15,24].

TWI and texture analysis (TA) belong to post-processing image analysis after nerve fiber reconstructions that involves another stage of analysis and calculation of the diffusion, after DWI acquisition, called constrained spherical deconvolution. TWI and TA evaluate the orientation and distribution of nerve fibers from DWI-MRI data. With the help of TWI maps, the details of the majority of the nerves in the head and neck region can be included. These post-processing techniques allow the surgeon to identify preoperatively the course and the relationships of the facial nerve. TWI allows calculating the FA values of the facial nerve. It has been demonstrated that lower FA values (threshold of 0.1 – high sensitivity for nerve-tumor contact detection) are recorded in the cases of contact between the facial nerve and the parotid tumor. TWI is currently available only with 3 Tesla MRI scanners [16,17,55]. Texture analysis is a computer-aided technique that involves detecting and quantifying mathematical models called texture features found in the gray-scale distribution of pixels in digital images. Gray-level histogram, the co-occurrence matrix, the run-length matrix, and the wavelet transform are the most commonly used texture features. With texture analysis, based on the T1 and contrast-enhanced sequences, Fruehwald-Pallamar et al. were able to differentiate PMA from WT [38]. Promising results in the field of oncology are expected from TA-MRI [17].

DKI involves obtaining MRI images based on the diffusion of water molecules into tissues and the microstructural features that appear as a result of the organization of water molecules. Kurtosis coefficients (K_mean_, K_rad_, K_ax_, and K_app_) and diffusion coefficients (D_mean_, D_rad_, D_ax_, and D_app_) are mostly used for DKI-MRI investigation. Shun et al. reported that FA, together with K_ax_, had a Se of 71.43%, a Sp of 95.78%, and an accuracy of 91.77% in detecting WTs [15,56].

H-MRS is based on the measurements of metabolite concentrations from tissues and organs to characterize metabolic changes that appear secondary to malignancy. The malignancy diagnosis using H-MRS is based on the increased levels of proliferation biomarkers, increased cell membrane turnover and cholinic compound levels [15].

## CT

**FIGURE 8:**
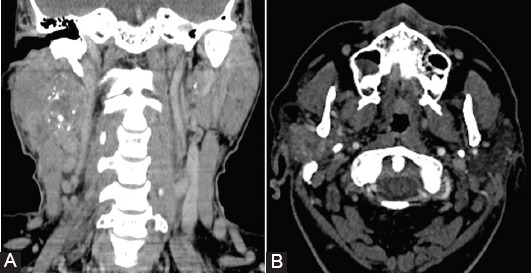
Contrast-enhanced computed tomography in a 65-year-old patient with histological proven salivary duct carcinoma of the right parotid gland. Multiple calcifications in the tumor lesion (A and B).

CT is an accessible cross-sectional imaging investigation, with a lower cost compared to MRI examination, but it involves exposure to ionizing radiation. CT provides an excellent resolution and contrast of the images, allowing a precise evaluation of parotid gland lesions in terms of location, size, density, presence of calcifications, and invasion of surrounding tissues (Figure 8).

CT has high accuracy in detecting salivary calculi and assessing tumor invasion of bone and cartilaginous structures [1,12]. However, compared to MRI, CT has a lower resolution of images for soft tissues which may lead to lower accuracy in the evaluation of parotid tumors. The main CT indications are contraindication of MRI examination, bone structure invasion, and assessment of cervical adenopathy [7,14,57,58]. CT can assess the stylomastoid foramen and the facial nerve position at the level of the mastoid bone. The limitations of the CT investigation are dental artifacts, contrast agent contraindications, and radiation concerns [10]. According to the results of a meta-analysis, CT has a Se of 83% and a Sp of 85% for the diagnosis of parotid gland tumors [13].

Dong et al. investigated the utility of contrast-enhanced CT in the differential diagnosis of parotid tumors. The results showed significant differences between time-density curve (TDC) values. PMA showed a gradually ascending TDC, WT showed a fast ascending TDC, followed by a fast descent, and malignant tumors showed a rapidly ascending TDC with a stable plateau [59].

## POSITRON EMISSION TOMOGRAPHY-CT (PET-CT)

PET-CT is based on the detection of radiation from the positron emission of a radiotracer administered to the patient. The radiotracer can be a glucose analog (fluorodeoxyglucose – most commonly used) that is absorbed in metabolic active cells, and with the help of CT the exact anatomical location is determined. PET-CT has a reduced role in the initial or differential diagnosis of parotid tumors due to low Se and Sp. However, PET-CT has a well-defined role in assessing regional, distant metastases, recurrent disease or second primaries, and in oncologic patient follow-up [1,7,10,12,58].

On PET-CT, WT may be hypermetabolic; therefore, during the oncologic patient follow-up, it may raise the suspicion of a metastatic or recurrent lesion [60].

Contrary to the majority opinion, Hadiprodjo et al. showed significant differences between fluorodeoxyglucose (FDG) uptake of malignant tumors compared to benign ones. Malignant tumors showed much higher values of maximum standardized uptake (SUVmax) and metabolic tumor volumes compared to benign tumors [61].

Wang et al. showed that performing whole-body FDG-PET-CT at the time of surveying the entire body condition can identify occult parotid tumors; 19% of the occult parotid tumors identified in his study were malignant [62].

MRI and CT investigations proved to also have a clinical utility for the evaluation of the minor salivary gland tumors. DWI and DCE-MRI sequences are useful for the diagnosis and characterization of tumor involvement and the detection of perineural invasion along the greater and lesser palatine nerves encountered in the cases of palatal tumors. The CT scan allows the minor salivary gland tumor diagnosis, identifies the possible adjacent bone invasions, and has good accuracy in differentiating neoplastic from non-neoplastic lesions [63].

Considering that 80–90% of parotid tumors are located in the superficial lobe, ultrasound (US) is often the first-line imaging investigation for parotid tumors diagnosis. US has high sensitivity and specificity in differentiating tumors from non-tumoral lesions but relatively low sensitivity in differentiating benign from malignant parotid tumors [64,65]. Modern multiparametric US techniques (shear wave elastography and contrast-enhanced US) complement the shortcomings of the conventional US, allowing a more complex evaluation of parotid tumors [64-67]. Multiparametric US provides promising results for parotid tumor differential diagnosis. The combined techniques of multiparametric US show high sensitivity and specificity (up to 91%) in differentiating parotid tumors. However, ultrasonography, due to its main disadvantages (operator-dependent technique, lack of standardized sections, difficulty in evaluating deep parotid lobe lesions or located posterior to bone structures), still cannot replace the MRI investigation [67,68].

## THE ROLE OF IMAGING IN THE POST-TREATMENT FOLLOW-UP OF PATIENTS WITH PAROTID GLAND TUMORS

MRI is considered the gold standard imaging examination for post-treatment monitoring of patients with parotid tumors. Routine MRI with DWI and DCE sequences is the preferred investigation for the detection of locoregional recurrences of benign and malignant parotid tumors. MRI can also detect with great accuracy the perineural spread of salivary cancers. An MRI investigation performed at 3 months after the end of treatment is indicated to facilitate the early identification of tumor recurrences. PET-CT is not superior to MRI in detecting the locoregional parotid tumor recurrences. MRI can differentiate postoperative modifications like scar tissue (low T2 signal and no contrast enhancement) from a recurrent tumor (intermediate signal on a T2 with moderate T1 contrast enhancement). DWI-MRI sequences can improve the differentiation of recurrence from post-treatment changes. For the detection of surgery-related complications of the salivary glands, a CT scan or even a US is sufficient. For the detection of distant metastases or second primary malignancy, PET-CT investigation offers the best results [9].

Patients undergoing head and neck radiotherapy may develop radiation-induced sialadenitis, which mainly affects the parotid glands. For the diagnosis of this form of sialadenitis, the MRI with DWI sequence is very helpful, revealing small parotid glands and lower SI on all sequences with intense contrast enhancement. The radiation-induced sialadenitis ADC values decrease significantly after radiotherapy [43].

## FNAB AND CORE NEEDLE BIOPSY (CNB)

Open biopsy is contraindicated in parotid gland tumors due to the risk of tumor cells spreading, facial nerve damage, or salivary fistula formation**.** The interest of head and neck surgeons for a preoperative cytological diagnosis is high [69]. FNAB has a high Se (96%) and Sp (98%) in distinguishing between neoplastic and non-neoplastic pathology.

The FNAB technique allows in most cases to obtain a cytological diagnosis after the puncture of the parotid masses and should be used before any surgical planning [7]. FNAB is a simple, low-cost method with a very low complication rate, easy to perform in an outpatient setting, and is accepted worldwide as a mean of preliminary diagnosis of parotid tumors [6,69]. US guidance of FNA can increase the accuracy of the investigation and is very helpful in deep lobe parotid tumors [7]. FNA involves puncturing the tumor with a 22-25G needle, followed by a few back-and-forth movements with or without use of aspiration. The harvested material is expressed on a microscopic slide [69].

Barats et al. showed in their study that an appropriate specimen must comprise of a minimum six-cell clusters per slide. Unsatisfactory aspirates (insufficient cell number or blood-only aspirates) can occur in up to 18% of cases [70].

According to the results of the reviewed studies, FNAB has a Se between 55.6 and 94.1%, a Sp between 75% and 98%, a PPV between 60% and 90%, a NPV between 86% and 98%, a non-diagnostic rate between 3% and 28.9%, and even 68% according to one author, and an overall accuracy between 60.8% and 98% [71]. The accuracy of FNAB in the diagnosis of tumor histological type is low (18–35%) [6,7,69,71-75]. The FNAB accuracy can be increased by examining the material immediately after sampling, by an experienced operator and interpretation of the result by a specialized pathologist and by using the US guidance; US guidance can avoid harvesting cytological material from tumor necrotic areas and confirms the presence of the needle tip in the center of the parotid tumor [72]. The cytological material aspirated with FNAB does not allow tumor grading, staging, or immunohistochemical analysis [72]. Fundakowski et al. have stated that the FNAB outcome can change the therapeutic decision in more than a third of cases; as a result, the cytological report must always be interpreted in a clinical context. In their study of 559 patients, the authors pointed out that indeterminate FNAB results must raise the suspicion of malignancy [73].

Major complications of FNAB such as dissemination of tumor cells or damage to the facial nerve are extremely rare; studies in the literature showed a risk of dissemination of only 0.00012%, not including the cases of facial nerve palsy. Factors that influence the risk of tumor cell spread are represented by the size of the needle, the number of passages through the tissues and the use of aspiration or capillary technique [69].

Finally, CNB is becoming more and more used in parotid gland tumors, especially in cases where FNAB is non-diagnostic. The CNB technique involves local anesthesia, a 17-20 G needle or automated spring-loaded biopsy device, and US guidance [7,71,72]. CNB allows the preservation of the histological architecture of the harvested tissue, which allows a great variety of anatomopathological and immunohistochemical investigations. Some authors claim that the tissue route of the needle left following the CNB must be excised during surgery, in the case of malignant tumors and PMAs [69]. In cases of inoperable malignant parotid tumors, CNB can provide enough material for diagnosis, thus avoiding the open biopsy [71]. Huang et al. concluded in their study that in cases with clinical suspicion of lymphoma the ultrasound-guided core needle biopsy (USCNB) technique should be preferred to US-FNAB because in most cases it can establish the diagnosis and avoid open biopsy [74].

CNB can cause minor complications such as hematoma (1.4–1.6%), bleeding, pain, and temporary facial nerve weakness due to local anesthesia. Dissemination of tumor cells or facial nerve palsy are extremely rarely encountered. CNB presents a series of limitations such as possible false-negative results, greater invasiveness than FNAB, and the need for local anesthesia [69,72].

According to the studies in the literature, CNB records better results in the diagnosis of parotid tumors, having a higher Se and Sp than FNAB in the diagnosis of malignancy, and most often allows tumor typing, grading, staging, and immunohistochemical analysis [64]. Regarding the specificity of differentiation between benign and malignant tumors, CNB and FNAB register close values [69]. The studies reviewed in this paper have shown that CNB presents the following values: Se of 92–96%, Sp of 95–100%, PPV of 100%, NPV of 90–99%, overall accuracy between 96% and 98.4%, and non-diagnostic rate from 1.6% to 4% [6,7,69,71,72,74].

The FNAB technique is indicated in all parotid tumors before surgical resection to establish a cytological diagnosis. CNB is indicated for parotid masses that are non-diagnostic on FNA, as well as for inoperable parotid tumors or without surgical indication such as hematologic malignancy (lymphoma), being able to establish a clear diagnosis.

The FNAB and CNB contraindications are represented by bleeding disorders, anticoagulant therapy, acute sialadenitis, skin infections, as well as purulent intraparotid collections [6,7].

For deep parotid tumors, with extension in the parapharyngeal space or close to vital structures, CT-guided biopsy techniques can be used. These involve placing an 18-19 G guide needle, CT-guided, at the periphery of the lesion followed by FNA or CNB. Sub-zygomatic, retromandibular, or transoral approaches with various trajectories guided by tumor location are the most commonly used [6,10].

**FIGURE 9:**
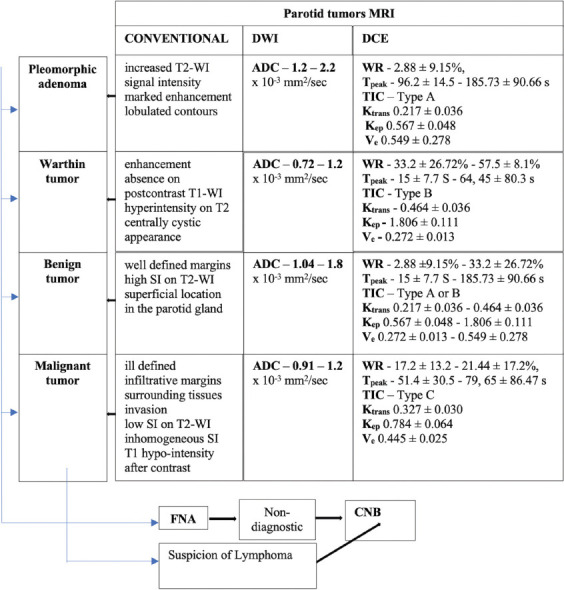
A guide for the preoperative differential diagnosis of parotid tumors using the most recent imaging and cytologic investigations.

A guide for the preoperative differential diagnosis of parotid tumors with the help of the most recent imaging and cytologic investigations, based on the reviewed studies is proposed in Figure 9 [1,4,5,12,15,17,19,20,22,25,26,29-32,36-38,45-50].

## LIMITATIONS

The limitations of the present literature review come from the fact that most of the existing and revised studies are retrospective, performed on small groups of patients. The radiologists or pathologists who have interpreted the investigation reports did not have a similar experience. Different statistical methods have been used in the revised studies. There are large variations between the imaging equipment and methods used and between the applied study protocols. Various cutoff values were used for analyzing the imaging parameters, which varied from study to study. In some studies, there was an unequal distribution between malignant and benign parotid tumors.

## CONCLUSION

For most clinicians, establishing an accurate differential preoperative diagnosis of parotid tumors is the main challenge, as it dictates the choice of therapeutic strategy. Modern imaging and cytologic techniques often allow the establishment of a preoperative differential diagnosis, if they are indicated correctly and are used according to a well-established protocol.

MRI is the first-line investigation used in the diagnosis of parotid gland tumors, being mandatory in parotid tumors that raise the suspicion of malignancy, larger than 3 cm and located in the deep parotid lobe or parapharyngeal space. Functional MRI is the imaging investigation of choice for the differential diagnosis of parotid tumors, with the highest Se, Sp, and accuracy, among the current imaging investigations.

Advanced MRI techniques alone or combined with functional MRI provide promising results for the differential diagnosis of parotid tumors. TWI-MRI allows the preoperative identification of the facial nerve course at the parotid gland level, being of great utility in planning the surgical intervention.

CT is indicated for the evaluation of parotid tumors that show bone or cartilage invasion, as well as when MRI is contraindicated or cannot be performed. For the evaluation of regional or distant metastases and for oncological patient follow-up, PET-CT investigation is most commonly indicated.

Performing US-guided FNAB by an experienced operator and analyzing the collected samples by a specialized pathologist allows a preoperative cytological diagnosis with high accuracy.

CNB is indicated when FNAB is non-diagnostic, as well as for inoperable parotid tumors or without surgical indication (parotid lymphoma), being able to establish a clear diagnosis.

Considering that the therapeutic decision and the prognosis of the patients with parotid tumors depend to a large extent on the preoperative diagnosis, combining cross-sectional imaging with cytologic investigations offers the most accurate results.
